# Permanent Draft Genome Sequence for *Frankia* sp. Strain CeD, a Nitrogen-Fixing Actinobacterium Isolated from the Root Nodules of *Casuarina equistifolia* Grown in Senegal

**DOI:** 10.1128/genomeA.00265-16

**Published:** 2016-04-07

**Authors:** Mariama Ngom, Rediet Oshone, Sheldon G. Hurst, Feseha Abebe-Akele, Stephen Simpson, Krystalynne Morris, Mame Ourèye Sy, Antony Champion, W. Kelley Thomas, Louis S. Tisa

**Affiliations:** a University of New Hampshire, Durham, New Hampshire, USA; b Laboratoire Mixte International Adaptation des Plantes et Microorganismes Associés aux Stress Environnementaux (LAPSE), Centre de Recherche de Bel Air, Dakar, Sénégal; c Département de Biologie Végétale, Laboratoire Campus de Biotechnologies Végétales, Faculté des Sciences et Techniques, Université Cheikh Anta Diop, Dakar, Dakar-Fann, Sénégal; d Laboratoire Commun de Microbiologie IRD/ISRA/UCAD, Centre de Recherche de Bel Air, Dakar, Sénégal; e Institut de Recherche pour le Développement (IRD), UMR DIADE, Montpellier, France

## Abstract

*Frankia* strain CeD is a member of *Frankia* lineage Ib that is able to reinfect plants of the *Casuarina* families. Here, we report a 5.0-Mbp draft genome sequence with a G+C content of 70.1% and 3,847 candidate protein-encoding genes.

## GENOME ANNOUNCEMENT

Members of the genus *Frankia* are soil-dwelling actinobacteria that are well known for their facultative lifestyle as a plant symbiont of dicotyledonous plants, termed actinorhizal plants (1–3). As ecologically important pioneer community plants, actinorhizal plants are found worldwide in a broad range of ecological and environmental conditions (4). The symbiosis allows actinorhizal plants to colonize harsh environmental terrains.

Based on molecular phylogenetic analysis, four major clusters within the genus are recognized (5–8) and genomes for representatives from each cluster have been sequenced (9–24). Cluster I contains two subclusters: One subcluster (cluster Ia) represents *Frankia* strains with the ability to infect a wider range of host plants including member of the *Betulaceae* and *Myricaceae* families, and the other subcluster (cluster Ib) contains strains limited to *Casuarina* and *Allocasuarina* host plants. Members of cluster II infect host plants of the subfamily *Dryadoideae* (*Rosaceae*), the families *Coriariaceae* and *Datiscaceae*, and the genus *Ceanothus* (*Rhamnaceae*)*.* Members of cluster III are the most promiscuous and are infective on *Eleagnaceae*, *Rhamnaceae*, *Myricaceae*, *Gynmmostoma* (*Casuarinaceae*)*,* and occasionally *Alnus*. The fourth *Frankia* lineage consists of the “atypical” strains which are unable to reinfect actinorhizal host plants or form ineffective root nodule structures that are unable to fix nitrogen.

Under tropic and subtropic conditions, fast growing and highly tolerant trees from the family *Casuarinaceae* have been used as windbreaks, dune stabilizers, fuel wood, and soil regeneration and these actinorhizal plants grow well under the harsh conditions including high salinity (25). *Frankia* sp. strain CeD was isolated from root nodules of *Casuarina equisetifolia* growing in Senegal and effectively reinfects its original host plant, *Casuarina* spp. (26). *Frankia* sp. strain CeD has been used extensively in infection studies and is well characterized for its host plant interactions (27).

The draft genome of *Frankia* sp. strain CeD was generated at the Hubbard Genome Center (University of New Hampshire, Durham, NH, USA) using Illumina technology (28) techniques. A standard Illumina shotgun library was constructed and sequenced using the Illumina HiSeq2000 platform, which generated 29,802,574 reads (260 bp insert size) totaling 4,381.0 Mbp. The Illumina sequence data were assembled using CLC Genomics Workbench (8.0.1) and AllPaths-LG (version r41043) (29). The final draft assembly for *Frankia* CeD consisted of 154 contigs in 120 scaffolds containing a total sequence of 5,004,600 bp with an *N*_50_ contig size of 73.6 kb and G+C content of 70.1%. This assembled draft resulted in 703× coverage of the genome.

The assembled *Frankia* sp. strain CeD genome was annotated via the Integrated Microbial Genomes (IMG) platform developed by the Joint Genome Institute, Walnut Creek, CA, USA (30, 31) and resulted in 3,847 candidate protein-encoding genes, 45 tRNA, and 2 rRNA regions.

### Nucleotide sequence accession numbers.

This whole-genome shotgun project has been deposited at DDBJ/EMBL/GenBank under the accession number JPGU00000000. The version described in this paper is version JPGU01000000.
